# 

**Published:** 1888-04

**Authors:** 


### TABLE OF CONTENTS.

ORIGINAL AND SELECTED ARTICLES.

Inflammation of the neck of the bladder, by Jno. Thad Johnson, M.D, Atlanta, Ga........ 121
Address of Judge Nisbet at the commencement exercises of the Southern Medical College, February 29, 1888.. ........ 126
Modern thought in medicine—Pres't Powell's charge to the graduates in medicine........... 131
Collinsonia canadensis ........ 136

ABSTRACTS AND GLEANINGS.

The treatment of intermittent fever........ 137
Bi-borate of ammonium in uric acid calculi.. 139
Treatment of sciatica; coca; guacal........ 140
Therapeutics of neuralgia of the head; the value of acetanilid in enteric fever........ 141
Pleuritis........ 142
How to preserve ligatures........ 143
Ergot in obstetric cases; toxic effects of iodoform, cutaneous and systemic........ 144
Ergot and acetic acid in post-partum hemorrhage ........ 145
The pupil as a guide in the administration of chloroform; saccharin........ 146
Quinine in gonorrhœa; the bichloride of mercury in the treatment of external diseases of the eye........ 147
Novel treatment of hemorrhoids; typhoid fever and coffee; a new remedy for night-sweats ........ 148
Diphtheria; intestinal obstruction........ 149
Tincture of iodine in persistent vomiting during labor; tar; cocaine in gonorrhœa; effects of tea........ 150

SCIENTIFIC ITEMS.

Warning apparitions; savagery in boyhood; The influence of forest upon rainfall and climate; a new gas........ 152

PRACTICAL NOTES AND FORMULÆ.

Glycerole for pigmentary spots on the skin; diarrhœa and dysentery; delirium tremens; indigestion; cough mixture........ 153
Cholera morbus of malarial fever; syphilis in the early stages; a new treatment for tape worm; fetid bronchitis; Prof. Da Costa........ 154

EDITORIALS AND MISCELLANEOUS.

Editorial brevities........ 155
A visit to the electric shaft........ 157
American Medical Association, May, 1888; County medical societies........ 158
Special Notes........ 160

